# Food allergen detection by mass spectrometry: the role of systems biology

**DOI:** 10.1038/npjsba.2016.22

**Published:** 2016-09-29

**Authors:** Derek Croote, Stephen R Quake

**Affiliations:** 1 Department of Bioengineering, Stanford University, Stanford, CA, USA; 2 Department of Applied Physics, Stanford University, Stanford, CA, USA; 3 Howard Hughes Medical Institute, Stanford University, Stanford, CA, USA

## Abstract

Food allergy prevalence is rising worldwide, motivating the development of assays that can sensitively and reliably detect trace amounts of allergens in manufactured food. Mass spectrometry (MS) is a promising alternative to commonly employed antibody-based assays owing to its ability to quantify multiple proteins in complex matrices with high sensitivity. In this review, we discuss a targeted MS workflow for the quantitation of allergenic protein in food products that employs selected reaction monitoring (SRM). We highlight the aspects of SRM method development unique to allergen quantitation and identify opportunities for simplifying the process. One promising avenue identified through a comprehensive survey of published MS literature is the use of proteotypic peptides, which are peptides whose presence appears robust to variations in food matrix, sample preparation protocol, and MS instrumentation. We conclude that proteotypic peptides exist for a subset of allergenic milk, egg, and peanut proteins. For less studied allergens such as soy, wheat, fish, shellfish, and tree nuts, we offer guidance and tools for peptide selection and specificity verification as part of an interactive web database, the Allergen Peptide Browser (http://www.AllergenPeptideBrowser.org). With ongoing improvements in MS instrumentation, analysis software, and strategies for targeted quantitation, we expect an increasing role of MS as an analytical tool for ensuring regulatory compliance.

## Introduction

Food allergy prevalence is rising^1^ and food allergies are now estimated to affect up to 8% of children and 5% of adults.^2^ The economic costs of food allergies have also grown: direct medical costs attributed to food allergy in the US have been estimated at $4 billion annually, which does not include an estimated $5 billion in annual out-of-pocket expenses and $14 billion in annual caregiver opportunity costs.^3^Although there has been progress in developing desensitization regimens^4,5^ and therapeutics,^6,7^ strict avoidance of allergenic foods is often the only management solution. Peanut, milk, egg, soy, wheat, fish, shellfish, and tree nuts are the ‘big 8’ major food allergens that must be labeled if intentionally added to a food in the US, Canada, Mexico, Australia, China, the European Union, and more.^8^ However, cross-contamination of allergens into unlabeled foods may still occur in shared production facilities, on shared equipment, or along the supply chain.^9^ In one European study, peanut was found in 25% of cookies and 43% of chocolate labeled with the precautionary phrase ‘may contain.’ More worrying was that 11% of cookies and 25% of chocolate without advisory labeling tested positive for peanut.^10^ In another example, the US Food and Drug Administration found evidence of milk in 75% of chocolate products with advisory labeling and evidence of milk even in products without advisory labeling or specifically with dairy-free claims.^11^ In some cases, precautionary allergen labeling is representative of the true risk; however, manufacturers are incentivized to apply such labeling liberally in attempt to avoid litigation.^12^ Some phrases have reached such ubiquity that parents of allergic children report ignoring them.^13–15^ More accurate labeling through quantitative allergen testing would improve quality of life for both allergic patients and their caregivers, but first analytical, institutional, and regulatory challenges must be overcome transparently and with accountability to the numerous stakeholders involved. Major steps are required to institute food industry allergen risk management strategies,^16^ develop an agreement regarding thresholds for clinical reactivity,^17,18^ and establish robust analytical workflows capable of accurate allergen quantitation.^19^ This review focuses on the last challenge.

## Methods of allergen quantitation

There are two established methods for quantifying allergenic protein in food. The most common, commercially available enzyme-linked immunosorbent assays (ELISAs), rely on monoclonal or polyclonal antibody recognition of one or more allergenic proteins in a food. Following protein extraction, these assays consist of a series of incubation and wash steps before sample concentrations are interpolated from a standard curve generated using a serial dilution of an allergenic protein standard. ELISAs report detection limits of ~0.1–5 mg kg^−1^, also reported as parts per million (p.p.m.);^20,21^ however, these values need to be considered within the context of clinical reactivity of allergic individuals. Dose thresholds have been estimated from meta-analyses of gold standard double blind placebo controlled food challenges,^22–24^ but such estimates are challenging^17,25^ and garner much debate.^26,27^ Nonetheless, a cautious lower bound appears to be hundreds of micrograms or greater for peanut, milk, soy, wheat, hazelnut, cashew, and more.^23^ Consequently, the reported sensitivities of ELISAs are thought to be sufficient for the majority of allergic individuals consuming reasonable serving sizes of food.^28^ Beyond being sensitive, ELISAs do not require high levels of expertise to use and the process of ELISA development is mature, with guidance for the use of naturally incurred standards^28^ and for validation.^29^

ELISAs suffer from a number of disadvantages, however. Although antibodies recognize specific protein epitopes with exquisite specificity, they can exhibit little to no sensitivity for foods subjected to thermal processing, which can denature or degrade epitopes.^30–32^ In other instances, significant homology between allergenic proteins can result in false-positives owing to antibody cross-reactivity.^33–35^ Furthermore, differences in antibody composition, target analyte(s), sample preparation procedures, and standards used for calibration between ELISAs can result in large quantitative differences when testing identical foods.^30,34,36–39^ Lastly, ELISAs cannot be easily multiplexed, which adds costs to food manufacturers routinely testing for multiple allergens.

The second method of allergen quantitation is liquid chromatography–mass spectrometry (henceforth MS), for which the key advantage is sensitive multiplexed quantitation of allergenic proteins. Proteins are first extracted from a food, then reduced, alkylated, and enzymatically digested into peptides. Trypsin is the most commonly used endopeptidase as its selective cleavage of proteins C-terminal to lysine and arginine generates peptides whose lengths generally fall within a range amenable to analysis by MS. This complex mixture of peptides is then temporally separated during liquid chromatography based on differences in relative affinity of the peptides for the column (stationary phase) and solvent (mobile phase). Eluting peptides are then ionized through electrospray ionization and subsequently interrogated by the mass spectrometer.

Although MS^E^^40^ and parallel reaction monitoring^41^ have been demonstrated as quantitative proteomic techniques, the most extensively used targeted technique is selected reaction monitoring (SRM), also known as multiple reaction monitoring (MRM). SRM has been widely used to quantitate dozens to hundreds of proteins in complex matrices such as tissue,^42,43^ plasma,^44^ and cell lysate.^45,46^ SRM is also the principal technique for allergen detection and quantitation.^36,37,47–55^ The triple quadrupole (QQQ) mass spectrometer underlies SRM, where the first and last quadrupoles act as static mass filters for a precursor ion and product ion, respectively, and the second quadrupole functions as a collision cell to fragment the precursor ion into product ions. Each target in an SRM assay is known as a transition and consists of the mass-to-charge (*m*/*z*) ratio of a precursor ion and one of its product ions. For each precursor ion, which is chosen to be a peptide with a +2 or +3 charge, 3–5 of its highest intensity product ions are selected. The fact that these 3–5 transitions are predefined means that the quadrupoles are non-scanning, resulting in one to two orders of magnitude better sensitivity and dynamic range over shotgun or directed techniques.^56,57^Although the limited number of targeted transitions has a specificity cost compared with full MS/MS scans where all product ions are measured, this is compensated for using criteria dictating transition co-elution, consistent transition peak area ratios, and interrogation of transitions only during a retention time window characteristic to each peptide.^58^ This last criterion, known as scheduling, is particularly beneficial, having been shown to increase SRM sensitivity, greatly increase the number of quantifiable peptides, and reduce interference from other isobaric precursor ions.^56,59^

SRM allergen detection studies report detection limits in the same 0.1–5 mg kg^−1^ range as ELISAs.^47,49,53,60,61^ However, the large dynamic range of protein abundance in complex matrices is an obstacle to further improvements in sensitivity. One potential avenue for achieving greater sensitivity is the immunoaffinity depletion of high abundance protein, as is common for SRM plasma biomarker quantitation.^62,63^ This could prove to be a useful avenue of further research, especially for foods characterized by a dominant protein-contributing ingredient; for example, depleting wheat protein in breads or cookies when quantitating milk proteins.

There are a number of differences between ELISAs and MS for quantitative allergen detection. For one, MS quantitates peptides deriving from allergenic proteins, in contrast to ELISAs, which quantitate the proteins themselves. The disadvantage for MS is that protein quantity is the clinically relevant metric and there are assumptions made in MS when calculating protein quantity from peptide signal intensity, as discussed later. On the other hand, quantitating peptides has the advantage of indifference towards conformational protein epitopes and thus MS may be more robust to certain forms of food processing. As multiple food allergens are often present in processing facilities and roughly 30% of children with food allergies have multiple food allergies,^64^ the capacity to quantitate numerous proteins simultaneously in MS is strongly appealing. ELISAs, on the other hand, report either total allergen content or require multiple assays for determining the quantity of multiple individual proteins. It follows that MS is also more robust to the composition of the input allergen. For example, milk has two dominant protein fractions, whey and casein, and both may not be present in a food. With a multiplexed method, MS is capable of quantifying these independently while ELISA results will be skewed based on antibody target(s). As we understand more about the specific protein alterations occurring during food processing, SRM assays can be easily augmented to include certain peptide modifications such as oxidation, deamidation, or glycation.^53^ In contrast, antibody generation can take months to years and cannot easily target these site-specific modifications.

In studies comparing both methods, the sensitivity and accuracy of MS have been shown to be either similar to ELISA^50,53,65^ or substantially greater depending on the ELISA, allergen, and degree of food processing,^36,37,61^ except in the case of pasta spiked with egg prior to cooking.^66^ MS is less mature, however, and although guidelines have been proposed for method development,^67^ allergenic protein and peptide targets are not standardized and a high level of expertise is required to develop targeted MS assays. In this review, we discuss quantitation of food allergens using targeted MS in the context of these issues.

## Allergenic protein and peptide target selection in MS

**Figure 1:**
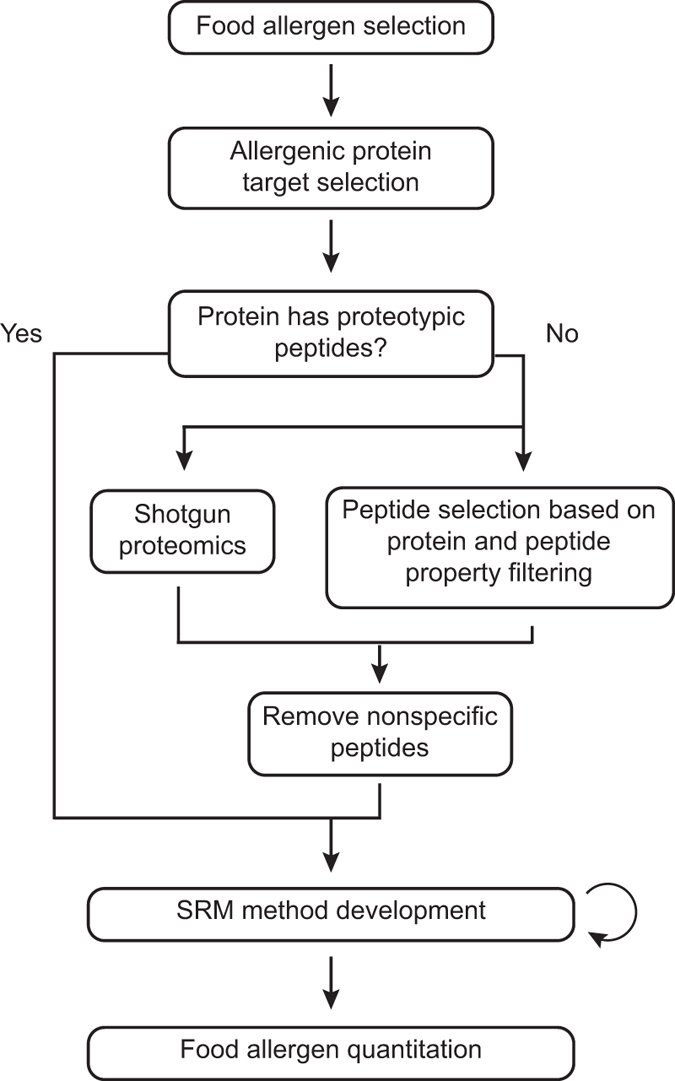
Workflow for allergen quantitation using MS.

Food allergen quantitation using MS is accomplished in four stages: protein and peptide target selection, peptide specificity verification, targeted method development, and quantitation. Selection of protein and peptide targets necessarily begins with the selection of one or more food allergens of interest as shown in Figure 1. For the big eight food allergens, which are responsible for a majority of allergic reactions, the proteins responsible for clinical reactivity are generally well understood^68,69^ and are often abundant within the food. For example, 94% of egg-allergic individuals were sensitized to egg white protein Gal d 2 (ovalbumin),^70^ which comprises 54% of egg white protein;^71^ 45–87.5% of peanut-allergic individuals were sensitized to Ara h 3 (an 11S globulin),^72,73^ which comprises ~30% of peanut protein;^74^ 85–100% of peanut-allergic individuals were sensitized to Ara h 2 (a 2S albumin),^72,75^ which comprises roughly 10% of peanut protein;^74^ and over 85% of milk-allergic individuals were sensitized to the dominant casein protein fraction in milk.^76,77^

In addition to abundance, there are further considerations for selecting protein targets. Ideally, proteins should exhibit consistent extraction from the food matrix, reproducible and complete digestion, and resistance to modifications during food processing.^67,78^ Furthermore, post-translational modifications, if present, should be well-characterized. Although this level of rigorous evaluation is currently absent for most combinations of allergenic protein, food matrix, and sample preparation scheme, some of these properties can be inferred by taking a systems approach in the form of a comprehensive assessment of the allergen detection literature, as we will describe. As for the number of proteins to include in the method, incorporating redundancy in the form of at least two proteins per allergen is important in order to mitigate the nonuniform effects of food processing, which include protein oxidation, glycation, denaturation, aggregation, hydrolysis, deamidation, and more.^53,79,80^ Clinically, these modifications do not necessarily eliminate reactivity and may even increase allergenicity,^79,81^ which reinforces the importance of assay redundancy for avoiding false-negatives and motivates further study of matrix-dependent protein modifications in the context of targeted MS.

We discuss three approaches to selecting target peptides for each allergenic protein: conventional shotgun proteomics, proteotypic peptide identification, and peptide filtering. A fourth approach could rely on public repositories of shotgun and targeted MS data such as the Global Proteome Machine,^82^ PRIDE,^83,84^ and SRM Atlas,^85^ but to date public MS data submission within the allergen detection and quantitation field has been low.

### 1. Conventional shotgun proteomics for protein and peptide discovery

Classically, in the absence of established proteotypic peptides, an undirected approach is used to identify peptides within an allergenic protein that can be utilized in targeted proteomics (Figure 1). Shotgun proteomics, also known as discovery proteomics, is applied to an allergen following protein extraction, reduction, alkylation, and digestion. The allergen, such as soy flour or egg powder, represents a possible contaminating ingredient in a food product and is preferably a reference material, as discussed later. Tandem mass spectra are acquired by a high-resolution instrument, such as a quadrupole time of flight or orbitrap mass spectrometer, operating in data-dependent acquisition mode.^57^ Subsequently, peptide sequences are assigned based on comparison of these spectra against a protein database using search algorithms such as Mascot,^86^ Sequest,^87^ or X!Tandem.^88^ Narrowing the list of identified peptides to those optimal for allergen quantitation then relies on selecting peptides with reproducible fragmentation profiles, strong signal intensities, and consistent retention times, while avoiding those with missed cleavage sites, amino acids prone to modification, or those which lack uniqueness to the target protein.^58,67,89^ Restricting the length of peptides to a maximum of 25 amino acids helps to ensure that the precursor and product ion *m*/*z* ratios fall within the mass analyzer’s operational range,^90^ while a minimum length of 7 amino acids is encouraged to improve the likelihood that the peptide is specific for the allergenic protein of interest. For allergens commonly subjected to food processing, it has also been suggested that C-terminal arginine peptides may be preferable to peptides with C-terminal lysine in order to minimize losses due to modifications such as the Maillard reaction;^53^ however, this suggestion requires additional empirical support.

### 2. Published literature suggests proteotypic peptides for certain major food allergens

**Figure 2:**
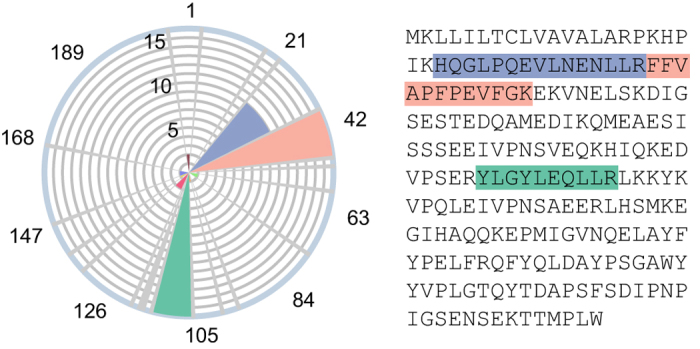
Bos d 9 (α-S1-casein) rose plot and protein sequence highlighting peptides HQGLPQEVLNENLLR (blue, 10 publications), FFVAPFPEVFGK (red, 16 publications), and YLGYLEQLLR (green, 16 publications) as proteotypic.

**Figure 3:**
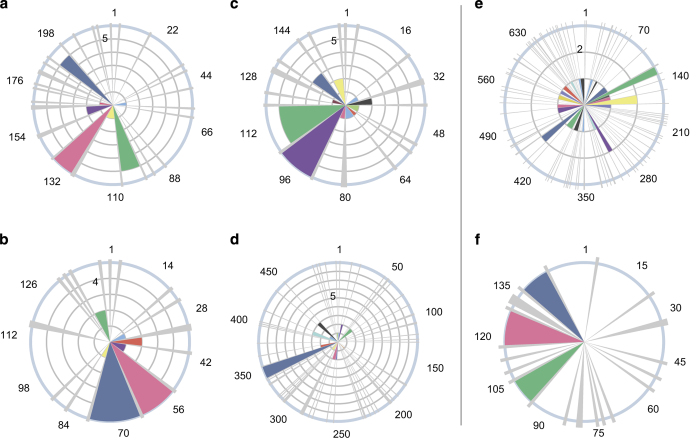
Variation in proteotypic peptide status among allergens. (**a**–**d**) Allergenic proteins with proteotypic peptides (itemized in Table 1). (**a**) Milk protein Bos d 10. (**b**) Egg protein Gal d 4. (**c**) Peanut protein Ara h 2. (**d**) Peanut protein Ara h 3.0101. (**e**, **f**) Examples of allergenic proteins with insufficient data for proteotypic peptide determination. (**e**) Egg protein Gal d 3. (**f**) Soy protein Gly m 8.

Many factors may affect the presence or intensity of peptides detected during MS, such as matrix component interactions, sample preparation protocol, liquid chromatography column and pressure, mass analyzer type, and experimental run mode. Consequently, it is not assumed that proteotypic peptides, which are peptides confidently detected in MS,^91^ exist that are robust to all of these potential factors. In light of this, targeted allergen detection studies perform shotgun proteomic experiments prior to any targeted assay development in order to first select peptide targets. This step is costly, time consuming, and generally reliant on commercial database searching software. Although such an undirected approach is worthwhile when the peptide targets are unknown, this step may be unnecessary for highly studied allergens. Specifically, if the same peptides for a given allergenic protein are detected in independent studies and in multiple matrices, there is the opportunity to standardize peptide targets using these proteotypic peptides, as has been previously suggested.^89^ To address this possibility, we aggregated published literature reporting the detection and/or quantitation of allergenic protein using MS and developed a rose plot for proteotypic peptide visualization, as shown for Bos d 9 (α-S1-casein) in Figure 2. The protein sequence, labeled by amino-acid position, spans the angular axis starting from the top and progresses in the clockwise direction. Each thin gray petal extending from the center of the plot to the radial maximum indicates a tryptic cut site, or in other words, a lysine or arginine in the amino acid sequence (except when followed by proline). Consequently, in between each gray petal is a tryptic peptide, colored so that they can be easily distinguished. The radial magnitude of each petal indicates in how many publications this peptide was reported. Therefore, this design highlights proteotypic peptides as radially large rose petals. In Figure 2, out of 11 potential tryptic peptides for the milk allergen Bos d 9 (α-S1-casein), the peptides HQGLPQEVLNENLLR (blue), FFVAPFPEVFGK (red), and YLGYLEQLLR (green) stand out as proteotypic, as these are reported in 10, 16, and 16 publications, respectively. Importantly, the studies from which these data are based vary in many or all of the aforementioned MS parameters, which only strengthens the assertion of these peptides as proteotypic. For example, these peptides have been detected in cookies,^49,53,65,61,92,93^ bread,^37^ mayonnaise,^94^ hazelnut spread,^95^ muffins,^36^ and wine^96–101^ following extraction with buffers based on urea/thiourea/SDS,^99^ ammonium bicarbonate/urea,^100^ acetonitrile,^53^ sodium bicarbonate,^95^ and Tris.^36,37,49,92–94,102^ For detection and/or quantitation, quadrupole time of flight,^65,92–94,100^ hybrid QQQ,^36,37,53,95^ ion trap,^49,61,96,99^ and orbitrap^97,98,101^ mass spectrometers have all been employed successfully. In addition to Bos d 9, milk protein Bos d 10 (α-S2-casein), egg protein Gal d 4 (lysozyme), and peanut proteins Ara h 2 and Ara h 3.0101 also contain proteotypic peptides, as shown in Figure 3a–d and listed in Table 1.

Ara h 3.0101 highlights one complicating issue in developing targeted methods using proteotypic peptides: the presence of isoforms of allergenic proteins, known as ‘isoallergens.’ Ara h 3 has two such isoallergens, Ara h 3.0101 and Ara h 3.0201, which share nearly 90% homology.^103^ A number of peptides are conserved between these isoallergens, but the proteotypic peptide SPDIYNPQAGSLK is only observed in the Ara h 3.0101 isoallergen. This suggests that for allergenic proteins with isoallergens, a combination of proteotypic and conserved peptides should be used to ensure specificity and sensitivity. It should also be noted that Ara h 2 also has two isoallergens, Ara h 2.0101 and Ara h 2.0201; however, these isoallergens are 93% identical and the identified proteotypic peptides are conserved between them. Although proteotypic assignment does not guarantee that a peptide is optimal for all food matrices and processing conditions, these examples of proteotypic peptides are encouraging for the future of MS as a reproducible allergen quantitation technique and highlight an appealing future direction for the field. Employing proteotypic peptides has the potential to eliminate the costs, time, complexity, and subjectivity of protein and peptide selection, while strengthening comparability between studies and facilitating assay standardization.

### 3. Selecting peptide targets for less-studied allergens

Not all allergenic proteins have sufficient data currently to support a set of proteotypic peptides. As shown for egg protein Gal d 3 (ovotransferrin, Figure 3e) and soy allergen Gly m 8 (2S albumin, Figure 3f), proteotypic peptides cannot be confidently assigned based on only three publications and one publication, respectively. Similarly, many soy, wheat, tree nut, fish, and shellfish proteins have insufficient evidence for proteotypic peptide assignment. Visualizations for these 100+ International Union of Immunological Societies (IUIS)-recognized allergenic proteins have not been included and are instead provided within our companion web tool, the Allergen Peptide Browser, available at http://www.AllergenPeptideBrowser.org/.

Selecting peptides for allergenic proteins lacking proteotypic peptides most often requires shotgun proteomics. However, for certain allergenic proteins, this may not be necessary, as depicted by the junction in Figure 1. The combined use of peptide filtering and detectability scoring can simplify later targeted method development by reducing the number of potential peptide targets for a given allergenic protein. The machine learning algorithms underlying detectability tools leverage the observation that tryptic peptides derived from the same protein can differ in signal intensity by orders of magnitude due to physiochemical differences such as hydrophobicity, charge, and size, as well as tryptic digestion efficiency.^104–106^

**Figure 4:**
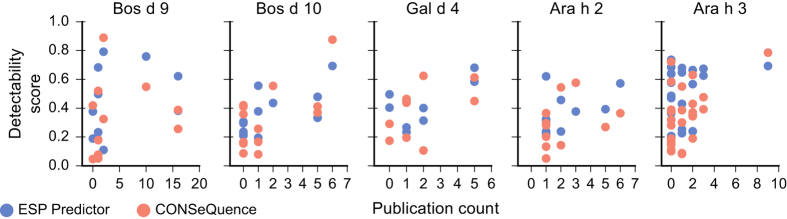
Comparison of peptide publication count with ESP Predictor and CONSeQuence peptide detectability scores for five allergenic proteins with proteotypic peptides. For Ara h 2, the Ara h 2.0101 isoform has been used, whereas for Ara h 3, the Ara h 3.0101 isoform has been used.

We compared the detectability scores from two popular *in silico* prediction algorithms, ESP Predictor,^107^ and CONSeQuence,^108^ with peptide publication counts generated from the aggregation of allergen proteomics literature, as shown in Figure 4. These tools do not show a strong ability to predict proteotypic peptides for the five allergenic proteins depicted in Figures 2 and 3a–d. Interestingly, there may be more utility in employing these tools to eliminate clearly non-proteotypic peptide targets; peptides with scores below a threshold of ~0.2 are reported in a minority of publications. The discrepancy between empirical and predicted detectability may be partially explained by the use of additional filtering criteria by researchers when selecting peptide targets. Examples of peptide attributes often avoided include potential glycosylation sites,^109^ consecutive cleavage sites,^110^ proline following an otherwise tryptic site,^111^ and certain amino acids that are prone to modification, such as cysteine or methionine.

The number of potential peptide targets can also be substantially reduced through the elimination of peptides that map to multiple allergenic proteins. For example, the walnut allergen Jug r 4 (an 11S globulin) has 21 tryptic peptides between 7 and 25 amino acids in length; however, many of these share specificity with other tree nut allergens. Eliminating these nonspecific peptides reduces the number of potential peptide targets to only 7. For smaller proteins such as 2S albumin allergens Jug r 1 (walnut), Cor a 14 (hazelnut), Ana o 3 (cashew), and Gly m 8 (soy), a comprehensive approach where all peptides are selected for initial method development is feasible given that these proteins contain on average only 9 tryptic peptides. However, if targeted method development will employ crude synthetic peptides as a first step in SRM method development, a decision discussed later, the costs associated with purchasing additional peptides should be considered. To aid in peptide selection, the Allergen Peptide Browser contains peptide detectability scores from ESP Predictor and CONSeQuence for all tryptic peptides, in addition to tools for filtering peptides by the aforementioned attributes.

## Peptide specificity verification

Prior to targeted method development, the specificity of each peptide must be assessed. This can be accomplished by querying each peptide sequence against public databases of nucleotide and/or protein sequence data, where nonspecificity is revealed by matches to species other than that of the allergenic parent protein. For example, an NCBI BLAST^112^ query of the non-redundant protein database for the peptide YMVIQGEPGAVIR reveals it is present in soybean allergen Gly m 3, rice allergen Ory s 12, and hazelnut allergen Cor a 2, among other fruit and food proteins. This indicates that the peptide is nonspecific and thus not suitable for allergen quantitation. For some allergenic proteins, nonspecificity may not be a concern. As an example, all tryptic peptides from milk protein Bos d 5 (β-lactoglobulin) map to milk from multiple species, but these species belong to either the cervidae (deer) family or the bovidae family, which includes such ruminants as bison, yak, cattle, and goats. In this case, either the highly homologous proteins from these species can elicit allergic reactions^113,114^ or the likelihood of a species being present in a food is sufficiently low that the nonspecificity of these peptides is acceptable. However, for the application of MS to food adulteration detection, such as the fraudulent addition of cow’s milk to goat’s milk,^115^ nonspecificity between similar species should always be considered.

To simplify the task of determining peptide specificity, BLAST results are included within the Allergen Peptide Browser for all tryptic peptides derived from allergenic proteins, with links to species and protein accession data hosted on NCBI. Furthermore, nonspecific peptides common to multiple allergens are clearly distinguished. Although this bioinformatic approach provides a simple and rapid method for avoiding nonspecificity, there is no guarantee that a peptide is specific due to inherent limitations of the known sequence space within databases. Continued sequencing efforts will only improve the comprehensiveness of these databases; however, the absence of a peptide should be confirmed in the allergen-free (blank) food matrix of interest during targeted method development. Of note, this is another instance where proteotypic peptides prove advantageous, as the numerous MS publications supporting proteotypic assignment contain this important negative control.

## SRM method development

Method development in MS requires a suitable input from which to derive appropriate peptide transitions and other assay parameters. A common, high-throughput approach to method development involves the interrogation of batches of up to ~100 crude, unlabeled synthetic peptides.^116^ These peptides are relatively inexpensive, eliminate the need to waste sample during development, are free from any matrix background, and yield quality spectra for peptides originating from otherwise low abundance proteins.^117^

Food allergens themselves, such as soy flour or egg powder, are alternatives to crude synthetic peptides for initial method development and offer a number of advantages. The use of an inexpensive and abundant source of target proteins free from an overwhelming background is a unique option for the allergen-detection field. In comparison, method development for low abundance plasma proteins contends with a high background of immunoglobulins, albumins, and other proteins and consequently requires synthetic peptides or recombinant proteins to derive SRM coordinates.^118^ Although the allergen must first undergo protein extraction and digestion, all target peptides should be present in high abundance, with the absence of a meaningful signal for any peptide strongly suggestive against its use for detecting trace amounts of allergen contamination in a food product. Furthermore, as the allergen has undergone sample preparation, the efficacy of amino-acid modification during the process, such as cysteine alkylation, can be faithfully evaluated. Allergens can be purchased locally at very low cost; however, locally sourced allergens will vary in moisture content, degree of processing, allergen content, and additional parameters, making comparisons between studies difficult. Reference materials (RMs) are a solution to this issue as their production carefully considers issues such as composition, homogeneity, and stability.^119^ Certified RMs (CRMs) are even more stringently developed, with certified values and uncertainties established from metrologically validated methods.^19^ CRMs are available for a number of allergens from the US National Institute of Standards and Technology, including whole milk powder (#1549a), peanut butter (#2387), and soy flour (#3234). However, RMs or CRMs do not yet exist for all allergens, in which case a commercially available or homemade version of an allergen can be used, for example, ground tree nuts.^120,121^ In such cases, the brand and properties of the input material along with all associated processing steps should be explicitly stated.

SRM method development requires the selection of transitions for each peptide target. An unbiased survey of potential transitions can be generated through the acquisition of full MS/MS spectra on a QQQ operating in SRM-triggered MS/MS mode using either synthetic peptides or an allergen tryptic digest.^116^ Unless the shotgun proteomics data were acquired on the same mass spectrometer to be used for quantitation, this approach is still prudent for studies employing shotgun proteomics given that peptide ionization, ion peak intensities, and fragmentation patterns may differ between instruments.^122,123^ The few input transitions that trigger full MS/MS acquisition can come from either shotgun proteomics data or from MS software. Skyline, an open source, vendor-neutral platform for targeted method development, facilitates this process by generating a transition list for each peptide through *in silico* digestion.^124^ For some instruments, Skyline is even capable of exporting the triggered method in native instrument format. After acquisition of the full MS/MS spectra, peptide sequences are assigned in the typical fashion using database search algorithms and according to established quality criteria.^125^ Results can then be imported back into Skyline where the peptides and transitions are ranked and can be subsequently filtered based on attributes described in the following paragraph. As an alternative to SRM-triggered MS/MS, Skyline can be used to generate a comprehensive set of expected high quality transitions that are interrogated during multiple unscheduled SRM runs.^123^ The tradeoff is that although database searching is avoided, more instrument time will be required due to the fact that all transitions are monitored during an unscheduled method as opposed to only monitoring the few that trigger full MS/MS acquisition during an SRM-triggered method. Depending on the number of target peptides, this may not be unreasonable; ~25 peptides can be monitored per run assuming an average peptide length of 14 amino acids, a 10 ms dwell time, and 2.5 s instrument duty cycle.^123^ The confidence in peptide identity will also be lower with this unscheduled approach as SRM coordinates have not been validated with a full MS/MS spectrum; however, the subsequent use of isotopically labeled peptides for quantitation is sufficient to validate SRM coordinates through retention time and relative ion peak intensity equivalence.^56^

SRM method refinement is an iterative process that enables the selection of robust assay parameters. First, the precursor ion charge state for each peptide must be selected. Unless the peptide is unusually long, the +2 charge state is generally chosen due to its greater signal intensity. For each selected precursor ion, selecting up to 10 of the most intense product ions is recommended for early stage refinement, with preference to y-ions over b-ions.^117,123^ Product ions should ideally have an *m*/*z* >5 above the precursor *m*/*z* to improve specificity, with the exception of special ions with known high intensities, such as those with N-terminal proline. Using the measured retention times for each peptide, a scheduled SRM method can be employed for subsequent injections. These replicates enable the evaluation of any drift in peptide retention time and the definition of an appropriate retention time window for each peptide. Furthermore, these replicates allow for the elimination of any unreliable peptides; however, as mentioned previously for allergenic protein, redundancy is an important characteristic of allergen quantitation and therefore at least two peptides per protein should be retained. In the final method, 3–5 of the highest intensity and most reproducible transitions per peptide are used for quantitation. Consequently, there will be at least 12 transitions monitored per allergen assuming 2 allergenic proteins per allergen, 2 peptides per protein, and 3 transitions per peptide. At this stage of method development, collision energy and declustering potential can also be optimized, although these steps are often considered optional due to increases in intensities of less than threefold^116^ and the fact that Skyline already includes instrument-specific equations that perform well in estimating collision energy.^126^

Lastly, SRM method development should consider the food matrix in which the allergen will be detected along with any processing the food may undergo. The effects of these two factors need to be empirically determined and therefore require the production of an incurred RM, defined as one in which the allergen is added to the food prior to processing.^28,127,128^ Examples of this include allergen spiked into cookie dough,^93^ wheat flour,^37^ and pasta flour before baking or cooking.^66^ As previously mentioned, the developed method should first be tested on the allergen-free RM prior to and following processing in order to ensure the absence of matrix-derived interference in any of the measured transitions. Next, the incurred RM should be evaluated following processing as the preferential loss of one or more peptides has been reported.^36,53^ Those peptides and transitions which are specific to the allergen and robust to processing are subsequently used for quantitation.

## Quantitation of allergenic proteins using SRM

Once an SRM assay has been developed, quantitation can be achieved using synthetic, stable isotope-labeled (SIL) peptides that behave identically to their unlabeled target analogues chromatographically and in fragmentation profile, but differ in mass.^45,129^ Termed AQUA,^45^ for ‘absolute quantitation,’ this approach has benefits of linearity over four orders of magnitude,^90^ coefficients of variation typically below 10%,^44,130^ and inter-laboratory comparability,^131^ all characteristics that are required for developing a standardized analytical workflow capable of quantifying allergens present in trace amounts in commercial foods. However, one disadvantage of using SIL peptides is that they cannot account for losses due to incomplete extraction and digestion of the target allergenic proteins. In contrast, an isotopically labeled protein can do so if spiked into the sample during the earliest stages of preparation.^53,67^ One study was able to correct for extraction efficiency using ^15^N-α-S1-casein;^53^ however, routine use of isotopically labeled proteins is prohibitively costly and thus quantitation using SIL peptides will suffer from some underestimation of protein abundance, although it should be noted that ELISAs also cannot account for such extraction losses. One interesting SIL peptide variation has been reported to improve quantitation by accounting for digestion variation. The authors used a SIL peptide with several amino acids added to both ends, which consequently requires cleavage at both bounding tryptic sites for release and detection.^132^ Although this elongated SIL peptide will not exhibit identical behavior to an endogenous protein, it may capture some food matrix and sample processing effects when compared against an SIL peptide, while costing less than an isotopically labeled protein.

Allergen quantitation using SIL peptides can be achieved with the following approach. Typically, the highest intensity peptide for each protein is used for quantitation, while other peptides are confirmatory. For both the endogenous and SIL peptides, the integrated peak areas for all transitions are summed,^133^ although others have used only the top performing transition. The ratio of endogenous to SIL area sum is then compared against a predetermined calibration curve.^129^ This calibration curve is generated by holding the SIL peptide concentration constant during a serial dilution of endogenous peptide.^53^ Importantly, high purity peptides should be used for calibration and the calibration curve should be matrix-matched, or in other words generated using a blank matrix. For sample quantitation, comparison of the measured peak area ratio against the calibration curve yields the amount of endogenous peptide, and thus, by best approximation, protein, on column. Depending on the desired form of the final reporting units, such as mg kg^−1^ of allergenic protein, corrections must then be applied which consider recovery estimates, amount of extractable protein in a food, protein molecular weight, and any dilutions during sample preparation.^36^ Finally, in demonstrating a novel SRM method, assays should also ideally report assessments of recovery, dynamic range, linearity, limits of detection, and limits of quantitation as part of a complete investigation into performance.^29,47,61^

Establishing inter-laboratory comparability for multiplexed, quantitative SRM with SIL peptides has been successful given the *a priori* selection of protein and peptide targets and their associated transitions.^131,134^ An analogous effort could standardize a multiplexed quantitative allergen workflow; however, this would similarly require predetermined SRM assay parameters. In this review we have demonstrated the existence of some proteotypic peptides from a systems approach and described how the use of these peptides can simplify the quantitative workflow. With regular submission of SRM assays into public repositories, the potential to standardize transitions in addition to peptides may also hold promise. We envision that once established, a SIL peptide panel could be widely employed and benefit the field in much the same way that synthetic spike-in RNA standards have improved the precision of RNA-seq quantification and increased experimental comparability across platforms and between laboratories.^135^

## Literature inclusion criteria

Published MS literature underlies the analysis of proteotypic peptides presented in this review. To be included, articles had to report the detection and/or quantification of IUIS-recognized allergenic proteins belonging to one of the big eight food allergens. Inclusion was further restricted to studies employing tryptic digestion and electrospray ionization for their applicability to a standardized and multiplexed quantitative workflow using SRM. A full list of the references is included under the reference section of the Allergen Peptide Browser.

## Conclusion

Unhelpful precautionary food labeling practices paired with growing worldwide food allergy prevalence motivates the development of an analytical workflow capable of multiplexed allergen quantitation in processed food. One step towards that goal involves establishing proteotypic peptides for the major allergenic proteins as targets within an SRM assay. Currently, however, proteotypicity falls along a spectrum. Food allergens such as wheat, soy, tree nuts, fish, and shellfish lie at one end of the spectrum, where insufficient published data necessitates further research before sets of proteotypic peptides are established. The Allergen Peptide Browser contains tools to enable accelerated targeted method development for these less-studied allergens, in addition to proteotypic peptide visualizations for all allergenic proteins belonging to the big eight food allergens. At the other end of the spectrum, ample empirical data demonstrates that proteotypic peptides exist for several key allergens, rendering shotgun proteomic experiments unnecessary prior to targeted method development. Importantly, the detection of these peptides appears robust to variation in food matrix, sample preparation protocol, and LC–MS instrumentation. Although allergen quantitation using MS has historically required high levels of expertise for method development, far less is required for routine analysis. Consequently, this review has sought to describe a targeted MS approach to allergen detection in which software and database advances can assist in simplifying and standardizing the MS approach.

## Figures and Tables

**Table 1:** Publication count and rose petal color corresponding to the proteotypic peptides depicted in Figure 3a–d

*Protein*	*Peptide*	*Rose petal color*	*Publication count*
Bos d 10	ALNEINQFYQK	Green	5
	NAVPITPTLNR	Pink	6
	FALPQYLK	Blue	5
Gal d 4	FESNFNTQATNR	Pink	5
	NTDGSTDYGILQINSR	Blue	5
Ara h 2	CCNELNEFENNQR	Purple	6
	CMCEALQQIMENQSDR	Green	5
Ara h 3.0101	SPDIYNPQAGSLK	Blue	9
